# Comparative Transcriptomics of *Bacillus mycoides* Strains in Response to Potato-Root Exudates Reveals Different Genetic Adaptation of Endophytic and Soil Isolates

**DOI:** 10.3389/fmicb.2017.01487

**Published:** 2017-08-04

**Authors:** Yanglei Yi, Anne de Jong, Elrike Frenzel, Oscar P. Kuipers

**Affiliations:** Molecular Genetics Department, Groningen Biomolecular Sciences and Biotechnology Institute, University of Groningen Groningen, Netherlands

**Keywords:** *Bacillus mycoides*, endophyte, root colonization, transcriptome, root exudates

## Abstract

Plant root secreted compounds alter the gene expression of associated microorganisms by acting as signal molecules that either stimulate or repel the interaction with beneficial or harmful species, respectively. However, it is still unclear whether two distinct groups of beneficial bacteria, non-plant-associated (soil) strains and plant-associated (endophytic) strains, respond uniformly or variably to the exposure with root exudates. Therefore, *Bacillus mycoides*, a potential biocontrol agent and plant growth-promoting bacterium, was isolated from the endosphere of potatoes and from soil of the same geographical region. Confocal fluorescence microscopy of plants inoculated with GFP-tagged *B. mycoides* strains showed that the endosphere isolate EC18 had a stronger plant colonization ability and competed more successfully for the colonization sites than the soil isolate SB8. To dissect these phenotypic differences, the genomes of the two strains were sequenced and the transcriptome response to potato root exudates was compared. The global transcriptome profiles evidenced that the endophytic isolate responded more pronounced than the soil-derived isolate and a higher number of significant differentially expressed genes were detected. Both isolates responded with the alteration of expression of an overlapping set of genes, which had previously been reported to be involved in plant–microbe interactions; including organic substance metabolism, oxidative reduction, and transmembrane transport. Notably, several genes were specifically upregulated in the endosphere isolate EC18, while being oppositely downregulated in the soil isolate SB8. These genes mainly encoded membrane proteins, transcriptional regulators or were involved in amino acid metabolism and biosynthesis. By contrast, several genes upregulated in the soil isolate SB8 and downregulated in the endosphere isolate EC18 were related to sugar transport, which might coincide with the different nutrient availability in the two environments. Altogether, the presented transcriptome profiles provide highly improved insights into the life strategies of plant-associated endophytes and soil isolates of *B. mycoides*.

## Introduction

The rhizosphere harbors an enormous pool of soil microorganisms and is considered as the ‘hot spot’ for microbial colonization and activity (Prashar et al., 2013). Some of the rhizobacteria even have the capacity of multiplying inside roots and begin an endophytic lifestyle (Hardoim et al., 2008). Rhizobacteria and plants naturally interact in various ways. Beneficial plant–microbial interactions in the rhizosphere can result in the promotion of plant health and development (Mark et al., 2005). A number of plant growth-promoting rhizobacteria (PGPR) have been commercially used as adjuncts to agricultural practice and show great promise (Glick, 2012). The mechanisms of PGPR and plant root interaction have drawn considerable attention (Kuiper et al., 2004; Santos et al., 2014; Pangesti et al., 2015). However, only a few studies on plants and their interaction with endophytic bacteria have been reported so far.

Endophytic bacteria are defined as bacteria capable of colonizing the internal tissue of the plant without harming the host or causing any disease symptoms (Mónica and Esperanza, 2006). It has been shown that many endophytic bacteria can promote plant growth with a similar mechanism as rhizobacteria, e.g., phosphate solubilisation (Wakelin et al., 2004), indole acetic acid production (Lee et al., 2004; Jasim et al., 2014), and siderophore production (Rosconi et al., 2013). A number of endophytic bacteria are able to prevent the deleterious effects of certain pathogens by producing antimicrobial compounds or inducing systematic resistance in the host plant (Gómez-Lama Cabanás et al., 2014; Kandel et al., 2017). The beneficial effects of endophytes are often more pronounced than those of many rhizosphere-colonizing bacteria (Hardoim et al., 2008). A large diversity of bacterial species have the ability to adopt an endophytic lifestyle. These comprise more than 200 bacterial genera from 16 phyla, with Actinobacteria, Proteobacteria, and Firmicutes being the most predominant genera (Malfanova et al., 2013; Jin et al., 2014). The rhizosphere-competent bacteria, including *Azospirillum, Bacillus*, and *Pseudomonas* genera, are most frequently reported as plant internal tissue residents. In fact, the endophytic bacterial diversity can be considered as a subset of the rhizosphere and/or the root-associated bacterial population (Santoyo et al., 2016). This implies that only a limited number of members of the rhizosphere-competent bacteria can succeed in becoming endophytic. This is due to several factors: (1) plants can selectively recruit certain beneficial rhizobacteria (Lakshmanan et al., 2013); (2) only those rhizobacteria that are well-adapted for living inside plants and simultaneously show an aggressive colonization ability can become endophytes. It has been reported that several *Rhizobium etli* strains were preferentially encountered as maize endophytes and the highly competitive strain Ch24-10 was the most tolerant to 6-methoxy-2-benzoxazolinone, a maize antimicrobial compound that is inhibitory to some bacteria and fungi (Rosenblueth and Martínez-Romero, 2004). *Pseudomonas* strains that were isolated from the endosphere are phenotypically distinct from *Pseudomonas* isolates obtained from the root surface as identified by their lipopolysaccharide pattern, cell envelope protein pattern, and other biochemical characteristics (van Peer et al., 1990). A comparative genomics study by Timm et al. (2015) showed that endosphere isolates of *P. fluorescens* have significantly more metabolic pathways for utilization of plant signaling compounds than rhizosphere isolates.

During initiation of root surface colonization by rhizosphere microorganisms, the first event in the interaction with plants is the encountering of root exudates (Shidore et al., 2012). Plant roots can exude up to 20% of fixed carbon into the soil. Amino acids, organic acids, sugars, aromatics, and various other secondary metabolites comprise the majority of the low-molecular-weight root exudates, whereas high-molecular-weight exudates primarily include polysaccharides and proteins (Brencic and Winans, 2005). Microbes can use these compounds as carbon- and energy sources for growth and development. Apart from being an exogenous nutrition, some molecules from exudates may act as signals to alter specific gene expression patterns in the microbe, which might influence its interaction with the host (Mark et al., 2005). For example, the plant-released molecules, including flavonoids, stachydrine, and trigonelline are recognized as signals for induction of nodulation genes in various legume-associated bacteria (Phillips et al., 1992; Janczarek et al., 2015). The citric acid in cucumber exudates was shown to recruit *B. amyloliquefaciens* SQR9 and to induce its biofilm formation (Zhang et al., 2014). Root colonization by endophytic bacteria is hypothesized to follow a similar process as described for rhizosphere bacteria. It has been shown that plant compounds from leaf macerates can activate a LuxR family transcription factor and its regulated genes of the cottonwood tree endophyte *Pseudomonas* sp. GM79 (Schaefer et al., 2016). Thus, molecules released from plants might play an important role in the plant–endophyte interaction.

*Bacillus mycoides* is a spore-forming, gram-positive bacterium belonging to the *B. cereus sensu lato* group. Among this group, *B. mycoides* is probably the least recognized and unresearched species, since it is in contrast to *B. cereus* and *B. anthracis* not pathogenic and does not have insecticidal activity as for instance *B. thuringiensis*. On agar plates, *B. mycoides* forms unique rhizoid colonies with chains of cells forming filaments projecting radially and curving to the left or the right (Di Franco et al., 2002). *B. mycoides* is ubiquitous in the soil and the rhizosphere. Some isolates have beneficial plant growth promoting (PGP) and biocontrol activity in various plants, including sunflower (Ambrosini et al., 2016), cucumber (Neher et al., 2009), and sugar beet (Bargabus et al., 2002). Our research showed that certain *B. mycoides* strains were able to endophytically colonize potato roots with potential PGP activity (unpublished data). However, the mechanisms of the beneficial interaction between the host and this endophyte are not known. Transcriptomics is a powerful approach to investigate plant–microbe interactions, but *in situ* transcriptomics of plant–microbe interaction models has limitations due to the imbalances of biomass ratios. As an alternative, root exudates or similar simplified systems are therefore used to study the bacterial response in the symbiotic interaction models. Root exudates were shown to have profound effects on gene expression patterns of rhizosphere bacilli including *B. amyloliquefaciens* FZB42 (Fan et al., 2012) and *B. amyloliquefaciens* SQR9 (Zhang et al., 2015). A transcriptome analysis conducted with the endophytic bacterium *Azoarcus* sp. strain BH72 and *Oryza sativa* root exudates, identified several genes required for rhizosphere competence (Shidore et al., 2012). Taghavi et al. (2015) used sucrose as sole carbon source to mimic the plant-associated lifestyle of *Enterobacter* sp. 638. Their transcriptome study revealed that the small RNA *csrABCD* regulon plays a role in the physiological adaptation and possibly mediates the shift between the free-living and a plant-associated endophytic lifestyle.

In this study, we compared the transcriptome profiles of an endophytic strain and a soil strain of *B. mycoides* in response to potato root exudates. The aim was to investigate differences in the genetic responses of the endophytic and the soil isolate and to decipher the endophytic determinants of *B. mycoides*. The colonization ability of the endophyte strain EC18 and the soil strain SB8 was tested by plant inoculation assays and *in situ* fluorescence microscopy monitoring. The whole genome of the two strains was sequenced and used as a reference for the transcriptome analysis. *B. mycoides* strain EC18 and SB8 were grown in the presence or absence of root exudates of potato plants, and differences in their gene expression profiles were analyzed by RNA-seq. Some genes with specific functionality showed a distinct trend between endophyte and soil strain and may be considered as key factors determining the endophytic lifestyle of *B. mycoides*.

## Materials and Methods

### Strains and Culture Condition

*Bacillus mycoides* EC18 was isolated from internal root tissue of the potato cultivar *Seresta* during the early flowering stage, grown on a practice field in a sandy soil at Wijster, the Netherlands. The soil strain SB8 was isolated from bulk soil of the same area. For isolation, samples were spread on Nutrient Broth agar plates (NB, 0.5% peptone, 0.3% yeast extract, 0.5% NaCl, 1.5% agar) supplemented with 0.1 mg/ml cycloheximide to inhibit fungal growth and incubated at 28°C for 24 h. The strains were identified by particular rhizoid colony morphology and 16S rRNA gene sequence comparison. After isolation, *B. mycoides* strains were grown in Luria-Bertani (LB, 1% tryptone, 0.5% yeast extract, 0.5% NaCl) or Brain Heart Infusion (BHI, Bacto^TM^, BD Biosciences, France) medium at 30°C with 200 rpm agitation. *E. coli* strain MC1061 was used for cloning and was grown in LB at 37°C with 220 rpm agitation. When needed, 50 μg/ml of kanamycin or 100 μg/ml of ampicillin (*E. coli*) or 4 μg/ml of chloramphenicol (*B. mycoides*) were added to the growth media.

### Construction of GFP- and RFP-tagged *B. mycoides*

The vector PYB was constructed by replacing the pAMß1 origin of replication (ori) of plasmid PATΔS28 (Namy et al., 1999) with the gram-positive temperature sensitive ori PWV01 from PAW068 plasmid (Wilson et al., 2007). Thus, the new vector PYB contains a PUC ori, a spectinomycin resistance gene, and a gram-positive temperature sensitive ori. A 1 kb fragment of the α-amylase gene was amplified from the genome of *B. mycoides* and digested with the restriction enzymes SacI and KpnI and ligated into the corresponding site of PYB resulting in the plasmid PYB_amy. The green fluorescent protein (GFP) and red fluorescent protein (RFP) genes were cloned into PYB_amy to obtain the vectors PYB_amyGFP and PYB_amyRFP. The plasmid PYB_amyGFP was transformed into *B. mycoides* EC18 and PYB_amyRFP was transformed into strain SB8 by electroporation as described previously (Yi and Kuipers, 2017) and colonies were selected on 100 μg/ml spectinomycin LB plates at 30°C. Transformants expressing GFP or RFP were selected by an Olympus MVX10 MacroView fluorescence microscope. Several bright colonies were inoculated in BHI liquid medium and grown at 37°C to achieve a single crossover recombination. The cultures were diluted and plated on a BHI agar plate with 100 μg/ml spectinomycin and incubated at 37°C. Successful GFP and RFP integration was verified by colony PCR. The expression of the fluorescent protein was analyzed by fluorescence microscopy (Nikon Eclipse Ti, Japan) equipped with a CoolsnapHQ2 CCD camera. Images with GFP- or RFP- fluorescent cells were taken using 450–490 or 560–600 nm excitation for fluorescence channels and an Intensilight light as phase contrast channel. Final pictures were generated by ImageJ software^1^ by merging the two channels.

### Root Colonization of the Endophyte and Soil Strains Observed by Confocal Laser Scanning Microscopy (CLSM)

The overnight culture of GFP-tagged EC18 and RFP-tagged SB8 were diluted 50 times and the strains were grown in BHI liquid medium at 37°C to the exponential growth phase. Cells were collected by centrifuging at 3000 × *g* and cell pellets were washed by 10 mM MgSO_4_ twice and resuspended in 10 mM MgSO_4_ to an OD_600_ of 0.65. The viable cells were counted by counting colonies on LB agar plate containing 100 μg/ml spectinomycin. The densities of EC18 and SB8 suspension were 1.2 × 10^8^ to 1.4 × 10^8^ cfu ml^-1^. The plant root inoculation protocol was modified from Prieto et al. (2011). Briefly, Chinese cabbage seeds were surface sterilized by soaking in 2–3% sodium hypochlorite and 70% ethanol for 2 min, respectively. Seeds were washed with sterilized water three times to remove the remaining disinfectant and germinated on 25% Hoagland medium solidified with 0.6% agar. After 7 days growing in a climate chamber at 25°C and 16/8 h light/dark photo-period, plant root was dipped for 45 min in bacterial suspensions of GFP-tagged *B. mycoides* EC18, RFP-tagged *B. mycoides* SB8, and a 1:1 mixture of the two strains. Plants were then transferred to solid Hoagland medium plates and incubated in a climate chamber at 25°C and 16/8 h light/dark.

For the visualization of the plant colonization by *B. mycoides*, fresh roots of cabbage plants were collected at 2 and 3 days after bacterial inoculation. The whole root was mounted on a glass slide and imaged using a confocal laser scanning microscope (Zeiss LSM 800, Carl Zeiss, Germany). Lasers at the wavelength of 488 and 561 nm were used to excite GFP and RFP, respectively. In order to visualize the endophytic colonization, a three-dimensional model that was rendered from about 60 optical sections at 1 μm intervals, was obtained with the Zen lite software (Carl Zeiss, Germany).

### Genomic DNA Extraction, Sequencing, and Annotation

*Bacillus mycoides* strains were grown overnight in BHI broth at 30°C, 200 rpm. One ml of overnight culture was centrifuged and the pellet was resuspended in 250 μl SET buffer (75 mM NaCl, 25 mM EDTA, and 20 mM Tris-HCl at pH 7.5). The cells were lysed with lysozyme at 37°C for 30 min. Then 10 μl of 10 mg/mL RNase was added and incubated 10 min at room temperature to remove RNA. After that, 25 μl SDS (10%) and 10 μl proteinase K (20 mg/ml) were added and the cells were incubated at 55°C for 30 min. The lysate was treated with phenol–chloroform and the water phase was used to recover DNA by isopropanol precipitation. Precipitated DNA was washed two times with cold 70% ethanol and finally dissolved in TE buffer (10 mM Tris-HCl, 1 mM EDTA, pH 7.5). The quality and quantity of the genomic DNA was checked with a Nanodrop (ND-1000, Thermo Scientific, United States) and a Promega fluorometer (Quantus, Promega, United States).

Purified genomic DNA was sequenced using the MiSeq sequencing system of Illumina (Zerbino and Birney, 2008), yielding 250 bp paired-end reads with a mean library size of 400 bp. *De novo* assembly was performed using Velvet (Zerbino and Birney, 2008). Prediction of protein-encoding regions and automatic functional annotation was performed using the Rapid Annotations using Subsystem Technology (RAST) server (Aziz et al., 2008). Genes for tRNAs and rRNAs were predicted with tRNAscan-SE (Lowe and Eddy, 1997) and RNAmmer server (Lagesen et al., 2007) respectively, with the prokaryotic default setting. Insertion sequences (ISs) were identified by BLASTN searches against the ISfinder database (Siguier et al., 2006). Prophage regions were identified using the PHAST web server (Zhou et al., 2011). Orthologous protein coding sequences in *B. mycoides* ATCC 6462, *B. mycoides* EC18, and *B. mycoides* SB8 were analyzed by the OMA (Orthologous Matrix) orthology database (Altenhoff et al., 2014). In addition, the functionality analysis of all the genes was performed by searching against protein domain databases, including GO (Harris et al., 2004) and COG (clusters of orthologous groups) (Tatusov et al., 2003).

### Root Exudate Preparation and Bacterial Treatment

For the collection of potato root exudates, tubers were rinsed with water to remove surface particles. Clean tubers were bathed in 2–3% sodium hypochlorite and 70% ethanol for 2–3 min, followed by five washing steps with sterile distilled water. The tubers were then sowed in autoclaved pots containing wet vermiculite and kept in a climate chamber at 26°C, with a photoperiod of 16 h of light and 8 h of dark for 2 weeks. When the shoots sprouted, the seedlings were transferred into beakers filled with 20 ml of autoclaved water. The potato tubers were fitted in small plastic baskets, which support the tubers being just above the water surface and the roots hanging in the water. The water containing the exudates was collected and the beakers were refilled with sterile water. Sample collection was performed for 7 days after transferring the seedlings. A 100 μl aliquot of each collection was taken and spread on an LB plate to check for contamination and the contaminated samples were discarded. The other samples were stored at -20°C until use. An overnight culture of *B. mycoides* was diluted to an initial OD_600_ of 0.05 in 90 ml LB medium supplemented with 10 ml of root exudates collected from potato plants. The same amount of Mili-Q water served as control. Ten milliliter of root exudates were selected, since no discernible effect on bacterial growth was observed at this level. The cultures were grown at 30°C, 200 rpm for 1 h and cells were harvested for RNA isolation.

### RNA Extraction

The total RNA was isolated with the Roche RNA isolation kit with some modifications. The cell pellets were flash-frozen in liquid nitrogen and resuspend in 400 μl TE buffer in 2 ml screw-cap tube. Then 50 μl of 10% SDS, 250 μl chloroform, 250 μl phenol and 0.5 g glass beads (0.5 μm) were added. The samples were homogenized using a Mini-BeadBeater (607, BioSpec, United States) for three 45 s cycles and were chilled on ice between cycles. Following the disruption, samples were centrifuged at 4°C and the supernatant was mixed with chloroform. After centrifugation at 4°C for 5 min, 500 μl of the upper phase were transferred to a fresh tube and 1 ml of lysis/binding buffer from the Roche RNA isolation kit were added. The mixture was transferred to filter and collection tubes and centrifuged 15 s at 8600 rpm. The filter tube was dried by an additional centrifugation step for 15 s at 8600 rpm. DNaseI solution was added and DNA was removed by incubation at 15–25°C for 20–30 min. After two washing steps with the wash buffer (Roche RNA isolation kit), the RNA was eluted with 50 μl elution buffer (Roche RNA isolation kit) and stored at -80°C before sequencing. The RNA quality was analyzed with the Bioanalyzer (Agilent Technologies, Wilmington, DE, United States).

### RNA Sequencing and Data Analyses

RNA sequencing was conducted at PrimBio Research Institute (Exton, PA, United States). The raw RNA-Seq reads were trimmed from the adapter sequences and mapped against the reference genome sequence of each strain. The RPKM (Reads Per Kilobase Million) table of the core genome of the three strains (4743 genes) was generated. Differential gene expression analyses were carried out with the web server pipeline T-REx (de Jong et al., 2015), in which the RPKM values of the *B. mycoides* strain grown in the presence of root exudates was compare with that grown in the absence of root exudates. The significance threshold was *p*-value < 0.05 and fold-change > 2 (“TopHits” in T-Rex). Functional categorization including GO, InterPro, KEGG and PFAM domains, was performed on the Genome2D web server^2^. The RNA-seq data from this study have been submitted to the NCBI Gene Expression Omnibus (GEO^3^) under the accession number GSE98148.

## Results and Discussion

### Root Colonization of Endophytic and Soil-Associated *B. mycoides* Strains

**FIGURE 1:**
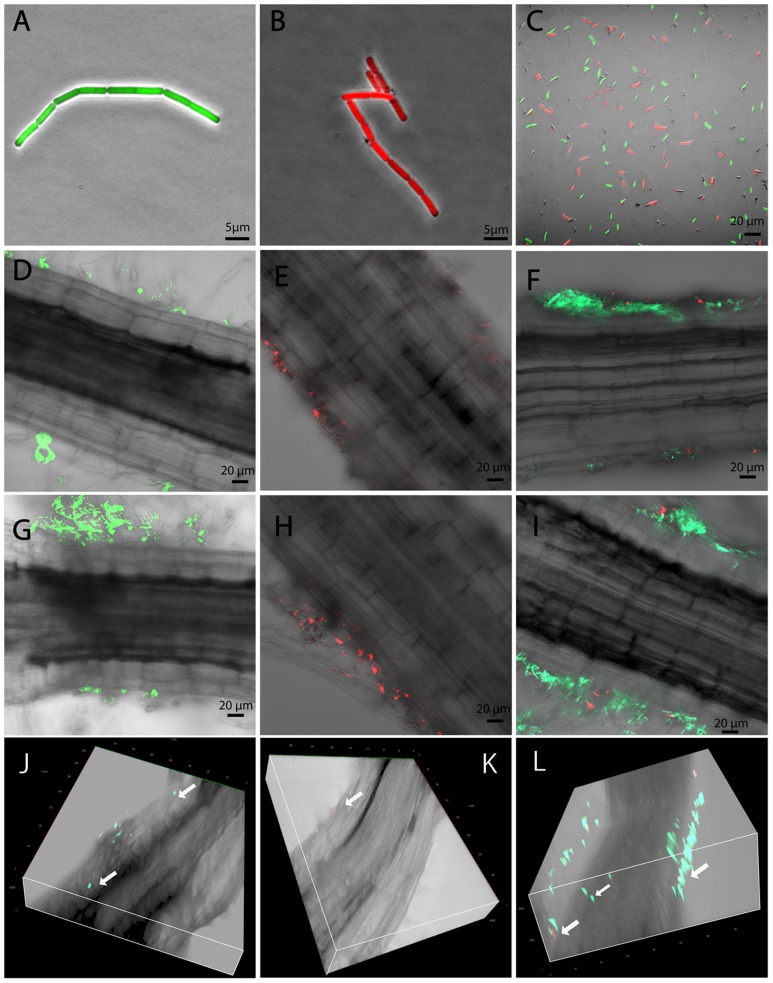
Root colonized by *Bacillus mycoides* observed by CLSM. GFP-tagged *B. mycoides* EC18 **(A)**, RFP-tagged *B. mycoides* SB8 **(B)**, and a 1:1 mixture of the two strains **(C)** were inoculated to the seedlings. Root samples were inspected 1 and 3 days after inoculation (DAI). **(D,G)** Root inoculated with *B. mycoides* EC18 at 1 and 3 DAI. **(E,H)** Root inoculated with *B. mycoides* SB8 at 1 and 3 DAI. **(F,I)** Root inoculated with a 1:1 mixture of *B. mycoides* EC18 and *B. mycoides* SB8 at 1 and 3 DAI. **(J**–**L)** Three dimensional model of roots inoculated with EC18, SB8, and a 1:1 mixture of the two strains 3 DAI.

In order to generate traceable *B. mycoides* strains for plant-interaction imaging experiments, the plasmid PYB_amyGFP was transformed into the endophytic *B. mycoides* strain EC18 by electroporation. A GFP gene controlled by a constitutive promoter was integrated in the α-amylase locus by curing the thermo-sensitive plasmid backbone at 37°C. A RFP gene was introduced into the strain SB8 with the plasmid PYB_amyRFP by appplying the same procedure. In order to compare the rhizosphere competence of the *B. mycoides* EC18 cells expressing GFP (**Figure 1A**) and of the SB8 cells expressing RFP (**Figure 1B**), a 1:1 mixture of the strains was inoculated into the hydroponic plant interaction model, and strain competition experiments were performed (**Figure 1C**). Inoculated cabbage plants were grown in a climate chamber and root samples were taken 1 and 3 days after inoculation (DAI). Subsequently, samples were examined for the presence of bacterial cells using confocal laser scanning microscopy (CLSM). When the strains were inoculated separately, both the endophytic strain *B. mycoides* EC18 (**Figure 1D**) and the soil strain *B. mycoides* SB8 (**Figure 1E**) were detected in the rhizosphere 1 DAI. Remarkably, when a 1:1 mixture of the EC18 and SB8 cells was inoculated, the roots were predominantly colonized by EC18 (**Figure 1F**). After 3 days of inoculation, colonization of the roots was progressing and the attachment of cells was significantly increased in the EC18 single inoculation group (**Figure 1G**). A similar trend was also observed for the SB8 population, albeit less significantly (**Figure 1H**). In the mixed inoculation group, the two strains shared a few colonization sites, however, the EC18 micro-populations predominated over the SB8 micro-populations (**Figure 1I**). A three-dimensional model showed that *B. mycoides* EC18 was able to colonize cortical tissues and xylem vessels 3 DAI (**Figure 1J**). In the mixture inoculation group, both strains were detected on the root surface, but only EC18 was detected inside the root tissues (**Figure 1L**). In the *B. mycoides* SB8 single inoculation experiment, endophytic colonization was not detected (**Figure 1K**).

As soil-borne microorganisms, several rhizosphere *Bacillus* species can enter plants and colonize internal tissues and many of them have shown PGP effects (Ji et al., 2008; Yi et al., 2013; Harun-Or-Rashid et al., 2017). The ability to colonize the rhizoplane is required for a successful endophytic colonization. As shown in **Figures 1D,E,G,H**, although both the EC18 and the SB8 isolates can establish a rhizoplane colonization, EC18 showed a more successful, faster and aggressive attachment. Following root attachment, the endophytic *B. mycoides* strain used a penetration process to enter the root interior and initiate the endophytic cell development. It has been discussed that some rhizosphere bacteria can occasionally and temporarily become endophytic, since passive penetration can take place at areas with damaged tissue, such as those occurring at root emergence sites or root tips (Compant et al., 2010). However, our data indicate that specific cell adaptations might be involved in active penetration of the root system by *B. mycoides*, because EC18 and SB8 colonized the same sites of the root, but only EC18 was able to enter the root tissue (**Figure 1L**).

### Genomic Analyses of *B. mycoides* Stains

**FIGURE 2:**
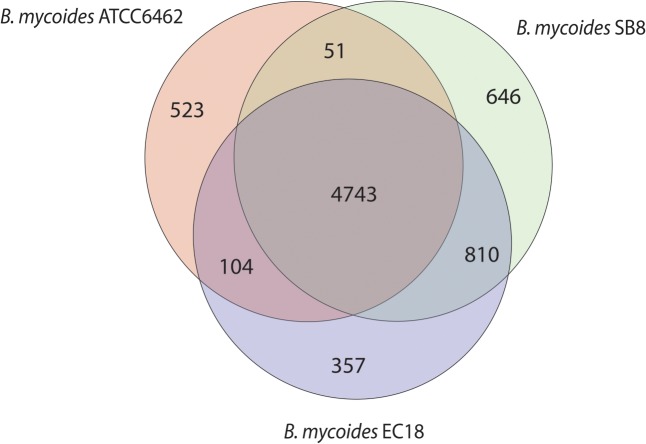
Venn diagram showing the core genome and the genes specific of the *B. mycoides* strain ATCC 6462, EC18, and SB8.

The Whole Genome Shotgun sequence of strains EC18 and SB8 has been deposited at GenBank under the accession MRWW00000000 and MRWS00000000, respectively. The general features of the genomes of EC18, SB8 and the reference strain ATCC 6462 are listed in **Table 1**. EC18 has a genome size of 5.75 Mb (Mega Base pairs) and SB8 has a genome size of 5.98 Mb, both of which are bigger than the type strain ATCC 6462 (5.64 Mb). The GC content of all three strains is around 35%. EC18 encodes 6,014 proteins, 77 RNA genes (7 rRNA and 70 tRNA genes) and 71 prophage-associated genes, while the SB8 genome comprises 6,250 protein-coding genes, 47 RNA genes (3 rRNA and 44 tRNA genes), and 177 prophage-associated genes. As shown in **Figure 2**, the core genome of the three strains consists of 4,743 orthologous groups and a pan-genome size of 7,234 genes, among which 357 genes are unique to EC18 and 646 genes are unique to SB8. 810 orthologous genes are shared by EC18 and SB8, 104 genes are shared between EC18 and ATCC 6462, while only 51 genes are shared between SB8 and ATCC 6462. The fact that SB8 and EC18 share 810 orthologous genes distinct from ATCC 6462, suggests that the latter strains are more closely related to each other than to the type strain isolate.

**Table 1:** A comparison of the genomic features of *Bacillus mycoides* strains ATCC 6462, EC18, and SB8.

	ATCC 6462	EC18	SB8
Genome size	5.64 Mb	5.75 Mb	5.98 Mb
GC content	35.4%	35.1%	35.1%
Protein encoding genes	5421	6014	6250
RNA genes	179	77	50
IS elements	22	42	47
Phage-associated genes	26	71	177

### Global Transcriptional Profiles in Response to Root Exudates

**FIGURE 3:**
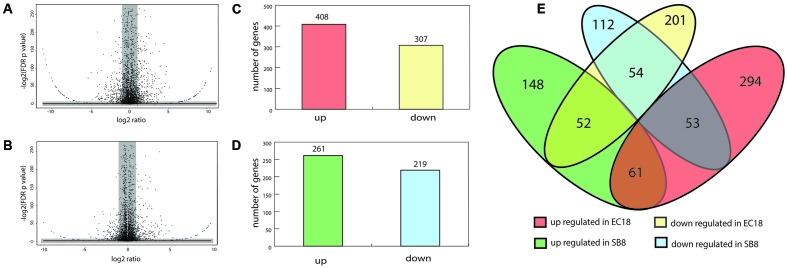
Analyses of differentially expressed genes (DEGs) of *B. mycoides* in response to potato root exudates. **(A,B)** Volcano diagrams of DEGs of EC18 and SB8. Spots in the right and left quadrant indicate significantly up and down regulated genes, respectively. Spots in the gray area were neither over- or under-expressed genes. **(C,D)** The number of up and downregulated DEGs of *B. mycoides* EC18 and SB8. **(E)** Venn diagram showing the numbers of overlapped and non-overlapped DEGs.

About 10 million reads were generated by RNA-Seq for each sample, of which 60–70% passed the quality filtering (Phred quality scores of > 20) using the FASTX-Toolkit version 0.0.13.2. Subsequently, the filtered reads were mapped on the genome of type strain *B. mycoides* ATCC 6462 to deduce the RPKM value of each gene or RNA. The RPKM values of the core genes (4,743 genes) were used to compare the transcriptomes of the strains under study using the T-REx pipeline (de Jong et al., 2015). Significant differentially expressed genes (DEGs) were shown in the TopHits genes close to cutoff values (*p*-value 0.05 and fold-change of 2) (**Figures 3A,B**).

**FIGURE 4:**
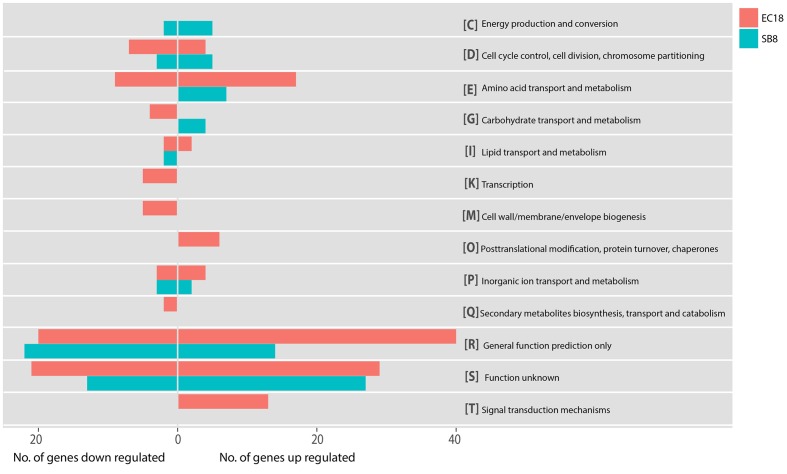
Functional categories of genes regulated of strain EC18 and SB8 according to COGs (Clusters of Orthologous Groups). Categorization of all altered genes was done on the Genome2D server (http://genome2d.molgenrug.nl).

In the endophytic strain EC18, 715 genes were significantly changed by the exposure to root exudates. Of those, 408 genes were upregulated and 307 genes were downregulated (**Figure 3C**). 193 out of the 715 altered genes were represented in the COG database and hence used to perform ortologanalyses. As shown in **Figure 4**, a large part of the genes are poorly characterized and belong to the group [R] (General function prediction only) or [S] (Function unknown). Apart from this, the upregulated genes were mainly implicated in amino acid metabolism and signal transduction, while many of the significantly downregulated genes were involved in cell cycle control, amino acid transport and amino acid metabolism, cell wall and membrane biogenesis, transcription, and carbohydrate transport and metabolism. The soil strain SB8 responded to the root exudates with an upregulated of 261 genes,whereas 219 genes were significantly downregulated. 109 of the in total 480 altered genes were found in the COG database. The largest group of altered genes were again clustered in [R] and [S], thereby leaving their function unknown or only providing an insight into a generally assumed function. The genes represented by COG classes with known function were mostly categorized in amino acid transport and metabolism, cell cycle control, and energy production and conversion. Taken together, the results showed clear differences in gene expression changes between EC18 and SB8 in response to potato root exudates.

### DEGs Showed Overlaps between Endophyte and Soil Isolate

**FIGURE 5:**
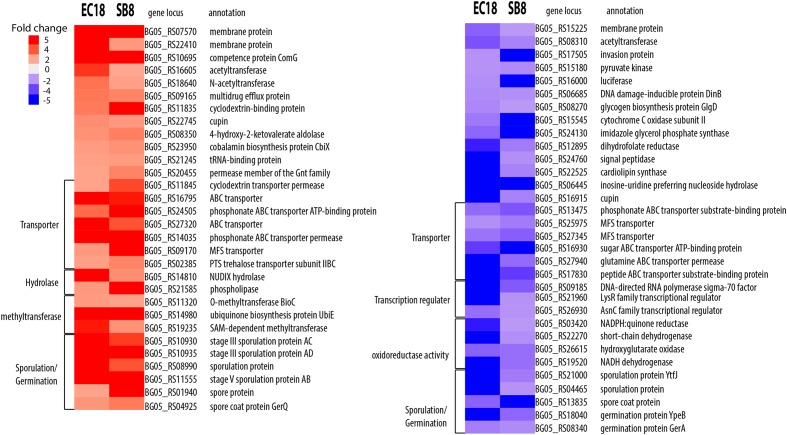
Overlapping DEGs of *B. mycoides* strain EC18 and SB8 in response to root exudates. Red represents significant upregulation and blue indicates significant downregulation of gene expression.

We next analyzed the DEGs that showed an overlap and similar changes in expression level modifications between the EC18 and SB8 isolates (**Figure 3**). In total, 115 genes were modulated in a similar pattern: 61 genes were upregulated and 54 genes were downregulated on both strains. While the expression of two membrane proteins was upregulated, the transcript levels were significantly lowered for a third membrane protein. Genes related to sugar transport (two genes), transcription regulators (three genes) and central intermediary metabolism (three methyltransferase genes) were also altered in the same pattern (**Figure 5**). One gene encoding a multidrug efflux protein (BG05_RS09165) was upregulated in both strains. It has been reported that multidrug efflux pumps are important for processes of detoxification of intracellular metabolites in plant host (Martinez et al., 2009). Similar studies with other rhizosphere-associated *Bacillus* species have shown that the sporulation and germination process can be affected by root exudates (Fan et al., 2012; Zhang et al., 2015). In line with these findings, nine sporulation and two germination genes were altered in the two strains in this study. This indicates that modulation of sporulation and germination pathways in response to root exudates is a response of *Bacillus* species independent from their ecological niche as a rhizosphere, endosphere, or soil strain. Furthermore, five genes with oxidoreductase activity were both downregulated by root exudates in the two strains.

### DEGs Showed Opposite Trends between Endophyte and Soil Isolate

A total of 105 DEGs showed a different pattern between EC18 and SB8. 53 genes were upregulated in EC18 but downregulated in SB8. On the contrary, 52 genes were downregulated in EC18 but upregulated in SB8 (??). The different expression profiles suggest that endophytic and soil-associated *B. mycoides* apply different genetic adaptation strategies to recognize or respond to plant-released signals. Presumably, The endophytic strain is able to change its gene expression pattern and thus adapt the metabolism toward a physiological state that enables an optimal nutrient acquisition, competition with species from the same niche and colonization of the plant.

**FIGURE 6:**
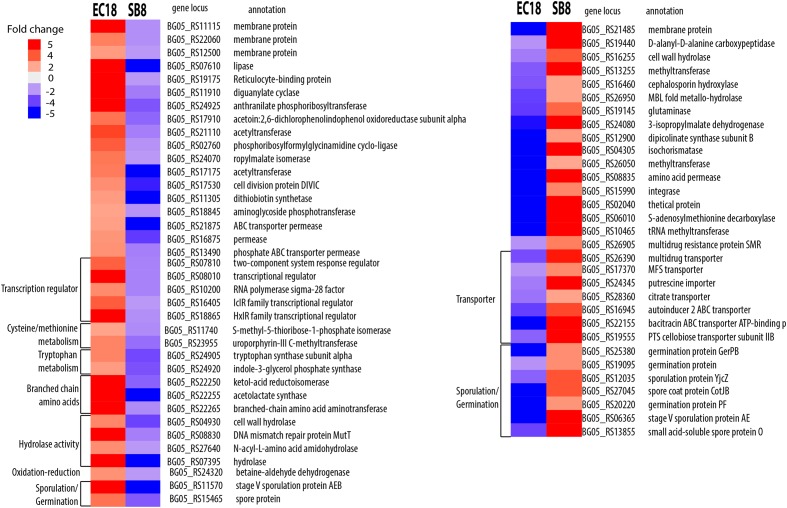
Differentially expressed genes show opposite trends in *B. mycoides* strain EC18 and SB8 in response to root exudates. Red represents significant upregulation and blue indicates significant downregulation of gene expression.

As shown in **Figure 6**, the genes being upregulated in EC18 and downregulated in SB8 included transcriptional regulators (five genes) among which IclR is related to multidrug resistance and degradation of aromatic compounds in soil bacteria (Song et al., 2004; Molina-Henares et al., 2006) and the sigma-28 factor is reported to transcribe the flagellin gene and control the transcription of a regulon specifying flagellar, chemotaxis, and motility functions in *B. subtilis* (Helmann and Chamberlin, 1987; Mirel and Chamberlin, 1989). Additionally, genes involved in the metabolism of amino acids showed an oppositely regulated expression pattern between the two strains. These included two genes involved in tryptophan metabolism (BG05_24905 and BG05_24920), three genes involved in branched-chain amino acid metabolism, e.g., BG05_22255 (*ilv*B), BG05_22250 (*ilv*C), and BG05_22265 (*ilv*E), and two genes involved in cysteine/methionine metabolism (BG05_11740 and BG05_23955). Xie et al. (2015) reported that several genes related to amino acids metabolism were stimulated when *B. subtilis* OKB105 was incubated with rice seedlings for 2 h. This finding is perhaps not surprising for rhizobacteria and endophytic bacteria, since amino acids are one of the major constituents of plant root exudates (Lugtenberg et al., 2001; Mark et al., 2005). However, it is interesting to see that those genes are downregulated in the soil strain when it encounters the root exudates.

Another group of genes was upregulated in the soil-derived strain SB8 and downregulated in endophytic strain EC18. These included two transporters involved in multidrug resistance proteins (BG05_26905 and BG05_26390) and several transporters including an major facilitator superfamily (MFS) transporter (BG05_17370), a citrate transporter (BG05_28360), a cellobiose PTS transporter (BG05_24345), and a putrescine importer (BG05_24345). Notably, a previous study has shown that the increased rate of putrescine uptake can decrease the competitive root colonization ability of *Pseudomonas fluorescens* WCS365 (Kuiper et al., 2001). The upregulation of this gene might thus decrease the rhizosphere colonization competence of SB8.

### DEGs Exclusively Changed in the Endophyte or the Soil Isolate

In addition to the overlapping transcriptional responses of the two strains, there is a large number of genes that were specifically modulated in one of the two strains studied. As shown in **Figure 4**, 495 DEGs were identified as endophytic strain-specific responses (294 up- and 201 downregulated) and 260 DEGs were identified as soil strain-specific responses (148 up- and 112 downregulated). Some of these genes have unknown functions and are currently annotated as hypothetical proteins (141 for EC18 and 91 for SB8). Altogether, this large number of specialized transcriptional changes underpins the different and specialized responses of endophytic and soil *B. mycoides* strain to the plant signals. We selectively discuss some genes that may participate in *B. mycoides*–plant interactions. In general, the endophytic strain showed a more complex response than the soil isolate. The DEGs are categorized based on GO, COG, and Pfam databases.

**FIGURE 7:**
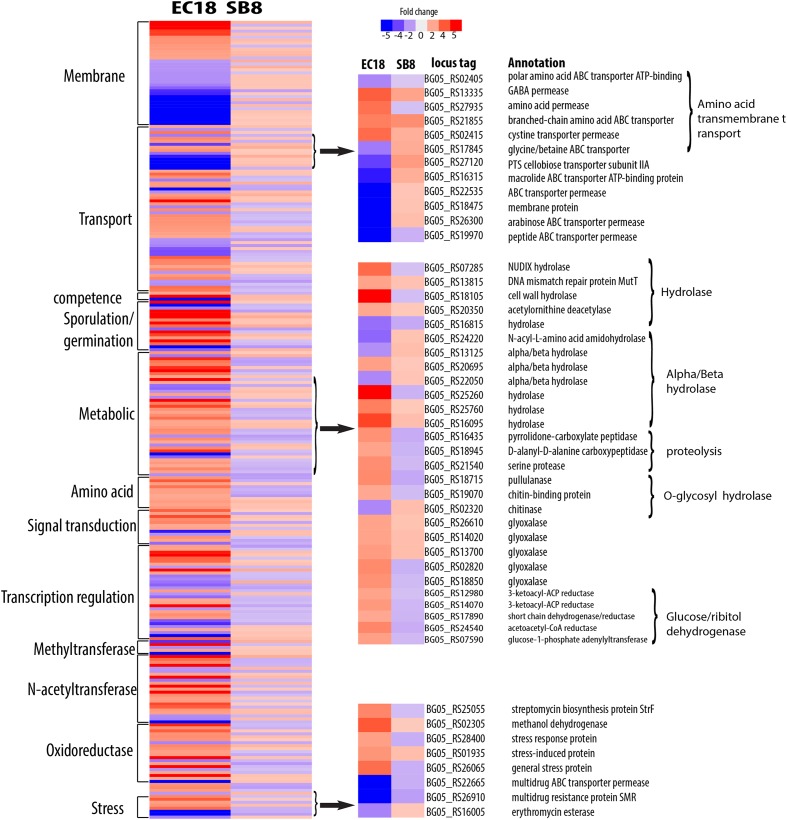
Differentially expressed genes specifically present in endophytic strain *B. mycoides* EC18 in response to root exudates. Red represents significant upregulation and blue indicates significant downregulation of gene expression.

**FIGURE 8:**
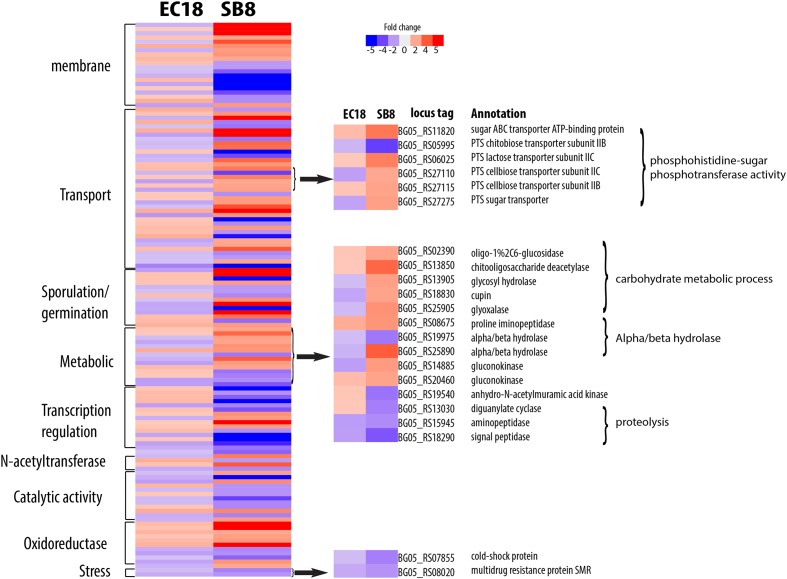
Differentially expressed genes specifically present in the soil strain *B. mycoides* SB8 in response to root exudates. Red represents significant upregulation and blue indicates significant downregulation of gene expression.

The endophytic strain responded to the root exudate with a significant upregulation of genes related to amino acid transmembrane transport, while genes involved in sugar transport including BG05_RS27120 (cellobiose PTS transporter IIA) and BG05_RS26300 (arabinose ABC transporter permease) were downregulated (**Figure 7**). However, as shown in **Figure 8**, several genes related to the PTS (phosphohistone-sugar phosphotransferase system) were significantly upregulated in the soil isolate SB8, i.e., those genes including sugar ABC transporter (BG05_RS11820), PTS lactose transporter subunit (BG05_RS06025), PTS cellobiose transporter subunit (BG05_RS27110 and BG05_RS27115), and PTS sugar transporter (BG05_RS27275). It was reported that some rhizosphere bacteria selectively and preferentially consume amino acids instead of sugars. The catabolite repression of glucose assimilation in the presence of amino acids was observed among the rhizosphere genera *Pseudomonas* and *Micromonospora* (Hoskisson et al., 2003; Moreno et al., 2009). Moreover, it has been shown that the enhanced ability to catabolize amino acids is responsible for the fitness gain during nutrient-limiting conditions (Zinser and Kolter, 1999). This suggests that bacteria, which are capable of modulating the amino acid uptake and preferring their utilization may have an selective advantage under such conditions. In this study, we observed that even within the same species, an endophytic strain and a soil strain show different metabolic preferences for amino acids or sugars due to the adaptation of the endosphere or soil niche.

A group of genes related to metabolic processes were specifically altered in the endophytic strain EC18 (**Figure 7**). These included four upregulated hydrolase genes (BG05_RS07285, BG05_RS13815, BG05_RS18105, and BG05_RS20350) and one downregulated hydrolase gene (BG05_RS16815). Additionally, four genes of the alpha/beta hydrolase fold protein family were upregulated and three downregulated in EC18. In comparison, only three alpha/beta hydrolase genes are altered in SB8 (two upregulated and one downregulated) (**Figure 8**). Proteins of the alpha/beta hydrolase fold family are involved in the degradation of plant cell wall polymers in *Burkholderia* spp. (Ali et al., 2014). Similarly, the expression of pullulanase (BG05_RS18715) and of the chitin-binding protein (BG05_RS19070) was upregulated in EC18. These two genes belong to the *O*-glycosyl hydrolase family and it might be possible that they are involved in the metabolism of plant-derived compounds. Three proteolysis genes including pyrrolidone-carboxylate peptidase (BG05_RS16435), D-alanyl-D-alanine carboxypeptidase (BG05_RS18945), and a serine protease (BG05_RS21540) were upregulated in EC18 (**Figure 7**), while all three proteolysis genes were repressed in SB8. Moreover, five glyoxalase genes were specifically upregulated in EC18 (**Figure 8**). Five genes encoding glucose/ribitol dehydrogenase (BG05_RS12980, BG05_RS14070, BG05_RS17890, BG05_RS24540, and BG05_RS07590) were upregulated in EC18. The overall metabolic gene expression profile suggests that the endophytic strain consumes a limited numbers of sugars (glucose/ribitol), while also using more types of other carbon substrates.

Among the genes related to stress response, three genes involved in multidrug resistance are downregulated in two strains, with two genes (BG05_RS22665 and BG05_RS26910) being more significantly altered in EC18 (**Figures 7**, **8**). A stress-induced protein (BG05_RS01935), a stress response protein (BG05_RS28400), and a general stress protein (BG05_RS26065) were specifically upregulated in endophytic strain. Genes encoding a methanol dehydrogenase (BG05_RS02305) and a streptomycin biosynthesis protein (BG05_RS25055) were also upregulated.

## Conclusion

Endophytic bacteria have emerged as a valuable source of biocontrol agents and metabolites, which has great potential in sustainable agricultural application. However, many aspects of the interaction between endophytic bacteria and plants are unclear, e.g., the endophytic colonization process and its differences compared to non-endophytic microorganisms. This study was designed to attribute the properties specific for an endophytic *B. mycoides* strain or its soil counterpart. Indeed, the CLSM observation showed that the endophytic strain is a more aggressive colonizer than the soil strain at an early stage of interaction with the plant root. The endophyte competes for the colonization site with the soil strain on the root surface and eventually penetrates the internal tissue of the root. It is presumed that the response of bacteria to environmental cues enables the gene expression pattern necessary for the endophytic life cycle. Our results suggest that root exudates of potato plants have a profound impact on gene expression levels in *B. mycoides* and that the endophytic strain showed more active or directed responses than the soil strain. The two strains showed a different metabolic preference when they encountered root exudates. The upregulation of genes related to amino acids metabolism, several proteolytic enzymes and *O*-glycosyl hydrolases points toward a specific adaptation to the ecological niche and a good rhizosphere fitness of the endophytic strain. In comparison, the nutrition source for the soil isolate seems to be constrained to sugars, which might hamper its colonization ability and proliferation rate in the rhizosphere. In conclusion, this study provides new insights into the different transcriptomic adaptations between an endophytic bacterium and its soil counterpart in response to potato root exudates and provides new knowledge on endophytic bacteria-plant interactions.

## Author Contributions

Conceived and designed experiments: YY, OK. Performed the experiments: YY. Performed GFP and RFP selection experiments: EF and YY. Performed bioinformatic analysis: YY and AJ. Wrote the paper: YY, OK, and EF.

## Conflict of Interest Statement

The authors declare that the research was conducted in the absence of any commercial or financial relationships that could be construed as a potential conflict of interest.

## Footnotes

1 https://imagej.nih.gov/ij/

2 http://genome2d.molgenrug.nl/

3 http://www.ncbi.nlm.nih.gov/geo/
